# Mechanical Stress Modulates the RANKL/OPG System of Periodontal Ligament Stem Cells via *α*7 nAChR in Human Deciduous Teeth: An In Vitro Study

**DOI:** 10.1155/2019/5326341

**Published:** 2019-05-02

**Authors:** Yujiang Chen, Kuan Yang, Zhifei Zhou, Lulu Wang, Yang Du, Xiaojing Wang

**Affiliations:** ^1^State Key Laboratory of Military Stomatology & National Clinical Research Center for Oral Diseases & Shaanxi Key Laboratory of Stomatology, Department of Pediatric Dentistry, School of Stomatology, Fourth Military Medical University, Xi'an, China; ^2^Department of Orthodontics, College of Stomatology, Xi'an Jiaotong University, China; ^3^Department of Stomatology, The General Hospital of Tibetan Military Region, China

## Abstract

The aim of this study was to investigate the mechanism by which periodontal ligament stem cells (PDLSCs) modulate root resorption of human deciduous teeth under mechanical stress. In this investigation, the PDLSCs were derived from deciduous and permanent teeth at different stages of root resorption. A cyclic hydraulic pressure was applied on the PDLSCs to mimic chewing forces in the oral environment. The cultured cells were characterized using osteogenic and adipogenic differentiation assays, quantitative real-time polymerase chain reaction (qRT-PCR), and Western blotting analysis. The PDLSCs exhibited the ability to induce osteoclast differentiation under certain mechanical stresses. As the expressions of RUNX2, alkaline phosphatase (ALP), and osteoprotegerin (OPG) were significantly reduced, the receptor activator of the nuclear factor kappa-B ligand (RANKL) was upregulated increasing the RANKL/OPG ratio. Under hydrodynamic pressure at 0-135 kPa, the expressions of alpha 7 nicotinic acetylcholine receptors (*α*7 nAChR), p-GSK-3*β*, and active-*β*-catenin were markedly upregulated in PDLSCs from unresorbed deciduous teeth. Treatment with the *α*7 nAChR inhibitor alpha-bungarotoxin (*α*-BTX) and the Wnt pathway inhibitor DKK1 may reverse the mechanical stress inducing upregulation of RANKL and reduction of RUNX2, ALP, and OPG. Alizarin red staining confirmed these results. The mechanical stress applied on the deciduous tooth PDLSCs can induce osteoclastic effects through upregulation of *α*7 nAChR and activation of the canonical Wnt pathway. It can be suggested that chewing forces may play a major role at the beginning of the physiological root resorption of deciduous teeth.

## 1. Introduction

The physiological root resorption of deciduous teeth is one of the most important physiological changes required for the shedding of deciduous teeth and eruption of permanent teeth [1]. The physiological root resorption of deciduous teeth is closely related to the genetically programmed odontoclastic activity [2] and further supplemented by alveolar bone remodeling, dominated by osteoblasts and osteoclasts [3]. The odontoclasts have ultrastructural characteristics, enzymatic properties, and metabolic properties similar to that of osteoclasts and play a main function in the resorption of the dental hard tissues (such as dentin and cementum) [4, 5].

The mechanism of root resorption is a complex multifactorial process and not fully understood. The physical pressure of erupting permanent teeth plays an important role in driving the root resorption of deciduous teeth [1]. The erupting force from permanent teeth affects corresponding deciduous teeth leading to root resorption [6]. Surprisingly, the roots of deciduous teeth typically resorb in the presence or absence of a permanent successor [7]. The periodontal ligament stem cells (PDLSCs) play an important role in the root resorption of deciduous teeth [8]. It has been reported that the receptor activator of nuclear factor kappa-B ligand (RANKL) may stimulate the formation of osteoclasts and odontoclasts, while the osteoprotegerin (OPG) regulates osteogenesis [9, 10]. The OPG functions as a secreted decoy receptor for RANKL. The RANKL/OPG ratio may indirectly reflect the conditions of the osteogenesis/osteoclastogenesis present in the microenvironment [11]. Li et al. reported that the RANKL/OPG ratio fluctuates during various stages of root resorption for primary teeth, whereas the PDLSCs modulate root resorption of deciduous teeth by regulating the expression of RANKL and OPG [8]. However, it is unclear how the expression of RANKL and OPG changes in the deciduous tooth PDLSC population.

Speculation regarding the development of jaw bones and muscles in children points to a gradual increase in the chewing forces applied on deciduous teeth, eventually exceeding the acceptable range of the periodontal ligament (PDL) [12, 13]. Additionally, the chewing forces may induce local production of cytokines that in turn stimulate the PDL fibroblasts to express RANKL and recruit macrophages and monocytes to activate odontoclasts leading to root resorption [14, 15]. Considering that the masticatory forces may play a role in the initial and subsequent stages of the physiological root resorption process, a number of researchers investigated the application of localized mechanical stress on PDLSCs and the resulting osteogenesis or osteoclastic activity [14, 16, 17]. For instance, Wei et al. [18] determined that the mechanical stress influences the expression of osteogenesis-relevant factors, such as alkaline phosphatase (ALP), RUNX2, and RANKL/OPG in human PDLSCs of permanent teeth. However, does the mechanical stress influence the RANKL/OPG system of PDLSCs in deciduous teeth? Further research is required regarding these mechanical stresses and their influence on osteogenesis/osteoclastogenesis and relevant factors of PDLSCs.

The alpha 7 nicotinic acetylcholine receptors (*α*7 nAChR) are capable of modulating osteoclastogenesis [19]. We have suggested previously that the periodontal ligament cells (PDLCs) regulate osteoclastogenesis through *α*7 nAChR [20]. Zhou et al. found that nicotine downregulated the osteogenic differentiation of the PDLSCs through *α*7 nAChR, which regulated the canonical Wnt pathway [21]. Furthermore, the Wnt pathway regulates the balance of the activity between osteoblasts and osteoclasts, thus maintaining bone homeostasis and participating in mechanical transduction [22]. In the process of mechanical transductions, the Wnt pathway provides conversion of extracellular mechanical signals into intracellular biological signals [23]. Similarly, during orthodontic tooth movements, mechanical stress regulates the osteogenesis/osteoclastogenesis differentiation of PDLSCs via the Wnt/*β*-catenin pathways [8]. Our previous study confirmed the existence of *α*7 nAChR in deciduous dental pulp stem cells (DDPSCs) and their regulation of the RANKL/OPG ratio during the process of root resorption [24]. The canonical Wnt pathway may regulate the expression of RANKL and OPG and change the RANKL/OPG ratio in DDPSCs [25]. However, the relationship of intracellular biological signal activation during the physiological root resorption process and the role of PDLSCs to modulate root resorption via the Wnt pathway is not well understood. The current study establishes a cell model to verify the hypothesis that the increased chewing forces by growing children apply more mechanical stress on PDLSCs of deciduous teeth, thereby regulating osteoclastic differentiation through *α*7 nAChR and the canonical Wnt pathway. The aim of this study was to investigate the mechanism of periodontal ligament stem cells that modulates root resorption of human deciduous teeth under the mechanical stress.

## 2. Materials and Methods

### 2.1. Tooth Samples

This study used permanent premolars extracted due to orthodontic treatment needs and retained deciduous teeth with healthy periodontium. Ethical approval was obtained from the institutional research ethics committee at the School of Stomatology, the Fourth Military Medical University, Xian, China.

**Figure 1:**
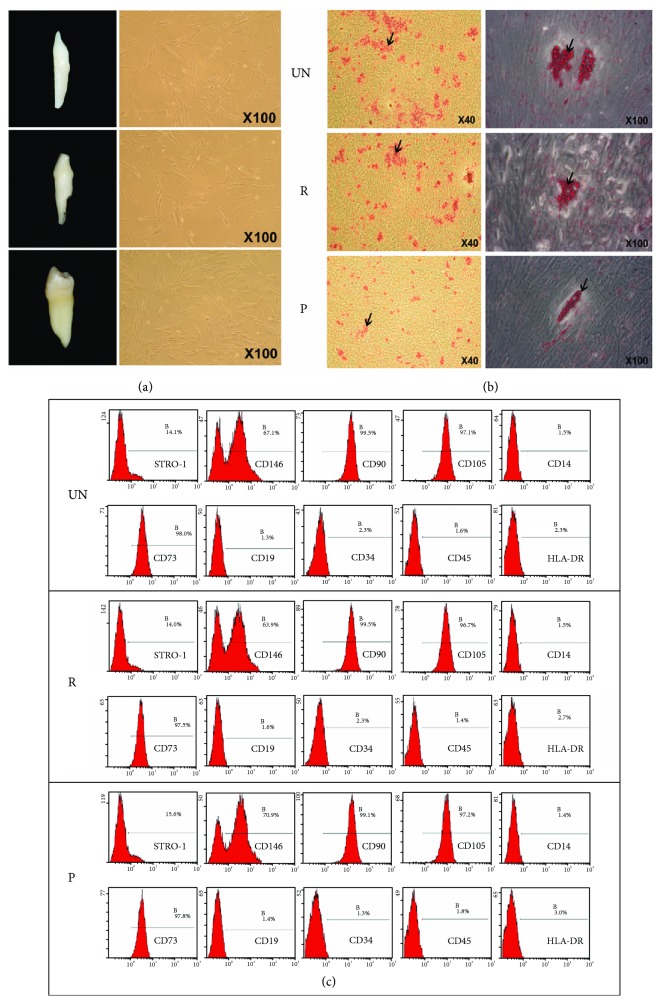
Isolation, culturing, and identification of PDLSCs. (a) Deciduous incisors and permanent premolars subdivided into three groups (UN, R, and P) showing the morphologies of the human-derived PDLSCs typical of fibroblast-like cells. (b) Cultured PDLSCs showing the mineralized nodules (alizarin red staining) following 21 days of osteogenic induction and lipid clusters (Oil red O staining) following 28 days of adipogenic induction. (c) Flow cytometry analysis of cultured PDLSCs from all groups revealed positive staining for STRO-1, CD146, CD90, CD105, and CD73 and a lack of staining for CD14, CD19, CD34, CD45, and HLA-DR.

Informed consent was obtained from every patient for use of their teeth for research purposes. According to the various stages of root resorption, the teeth were divided into three study groups (Figure 1(a)): the unresorbed group (UN): deciduous teeth without any root resorption (*n* = 6); the resorbed group (R): deciduous teeth showing root resorption (1/3 to 2/3 of the root length) from the lingual surface (*n* = 6); and the permanent tooth group (P): permanent premolars without any root resorption (*n* = 6).

### 2.2. Cell Culture

In order to remove all debris, teeth from each group were washed using phosphate-buffered saline (PBS) at room temperature. The periodontal ligament (PDL) from the middle third of the root was gently scraped using a sterile scalpel. The PDL tissues were dissociated for 15 min using type 1 collagenase (Sigma-Aldrich, Santa Clara, CA, USA) in an incubator at 37°C. Any residual undissociated tissues were removed using a 70 *μ*m cell strainer (BD Falcon, Bedford, MA, USA). The filtered, dissociated PDL tissues were neutralized with culture medium (alpha minimum essential medium (alphaMEM; HyClone, Logan, Utah, USA) containing 10% (*v*/*v*) fetal bovine serum (FBS; HyClone, Logan, Utah, USA) and 1% (*v*/*v*) penicillin/streptomycin (Invitrogen, Carlsbad, CA, USA)), followed by centrifugation at 1000 rpm for 5 min. The cell pellets were resuspended in the culture medium and incubated at 37°C with 5% CO_2_ until the cells surrounding the explants reached the confluence level. The culture medium was changed every 72 hours. Then, the cell layers were harvested by 0.25% trypsin-EDTA (ethylenediaminetetraacetic acid) solution (pH = 7.0–7.6, Sigma-Aldrich, Santa Clara, CA, USA). The limiting dilution technique and expansion was used to obtain a single cell colony. The second fourth passages of multiple colony-derived PDLSCs were used in the following experiments.

### 2.3. Osteogenic and Adipogenic Differentiation Assays

The osteogenic and adipogenic differentiation of PDLSCs was performed according to the methods reported previously [18–23]. Briefly, the PDLSCs (third passage) were placed in 6-well plates and suspended in culture medium until achieving ~90% confluency. Afterwards, the PDLSCs were incubated either in osteogenic medium (100 nM dexamethasone, 5 mM *β*-glycerophosphate, and 50 mg/ml ascorbic acid; Sigma-Aldrich, Santa Clara, CA, USA) for 21 days or in adipogenic medium (0.5 mM hydrocortisone, 0.5 mM methylisobutylxanthine, and 60 mM indomethacin; Sigma-Aldrich, Santa Clara, CA, USA) for 28 days. In order to identify the osteoblast and adipocyte-like cells, the standard alizarin red and the Oil red O staining were used, respectively. For the quantitative determination of the mineralized nodules, the cells were washed three times with PBS until the alizarin red solution and debris were removed. The prepared cells were observed under an inverted microscope and imaged. The presence of mineralized nodules was determined using Image-Pro Plus 5.0 software (Media Cybernetics, Silver Spring, MD, USA).

### 2.4. Immunophenotype Analysis

In order to identify the phenotype of PDLSCs, the cells were treated with surface markers and analyzed using flow cytometry. Briefly, the third-passage cells from each group were harvested by trypsin and washed using PBS (containing 1% bovine serum albumin). Cells were incubated in the dark at 4°C for 30 min with FITC-conjugated antibodies against STRO-1 (BioLegend, San Diego, CA, USA), CD14 (eBioscience, San Diego, CA, USA), CD34 (eBioscience, San Diego, CA, USA), CD45 (eBioscience, San Diego, CA, USA), CD73 (eBioscience, San Diego, CA, USA), CD105 (eBioscience, San Diego, CA, USA), and HLA-DR (eBioscience, San Diego, CA, USA) and PE-conjugated antibodies against CD19 (eBioscience, San Diego, CA, USA), CD90 (eBioscience, San Diego, CA, USA), and CD146 (eBioscience, San Diego, CA, USA). Cells were then washed and suspended for further characterization using flow cytometry (Coulter EPICS XL-MCL, Beckman Coulter, Fullerton, CA, USA). The isotype-matched normal IgG antibodies were used as controls.

### 2.5. Experimental Application of Mechanical Stress

A self-designed hydraulic pressure-controlled cellular strain unit was used to simulate chewing forces on the cultured PDLSCs and imitated the mechanical circumstances *in vivo* [26–29]. The hydrodynamic pressure compressing the gas phase (3.5% CO_2_, 96.5% N_2_) with a fixed frequency in the pressure chamber generated stresses on cells under investigation. PDLSCs from each group seeded in 6-well plates were further subdivided into five groups (*n* = 3). The four experimental subgroups were exposed to cyclic hydraulic pressure with a fixed frequency of 0.1 Hz at 0-45 kPa, 0-90 kPa, 0-135 kPa, or 0-180 kPa for 2 hours/day for three consecutive days. In addition, the hydraulic pressure loading was performed for 21 days consecutively during alizarin red staining experiments. During the stress loading process, the hydraulic pressure reached the peak gradually and then returned back to 0 kPa (frequency of 0.1 Hz). The control group was placed in an incubator at 37°C with 5% CO_2_, at standard pressure.

### 2.6. Alpha-Bungarotoxin (*α*-BTX) and DKK1 Treatment

The PDLSCs were treated with an *α*7 nAChR inhibitor, alpha-bungarotoxin (*α*-BTX; Tocris Bioscience, Bristol, UK), or Wnt signaling pathway inhibitor, DKK-1 (PeproTech, Suzhou, China), as described previously [25–27]. Briefly, the PDLSCs were trypsinized, counted, and seeded into 6-well culture plates at 5 × 10^4^ cells/well. The *α*-BTX (10^−8^ M) or 10 ng/mL DKK1 was added to the culture medium until the cells reached confluence. The cells were subsequently processed using a cyclic hydraulic pressure with 0.1 Hz frequency and 0-135 kPa/2 h for 3 days.

### 2.7. Total RNA Extraction and Quantitative Real-Time Polymerase Chain Reaction (qRT-PCR) Analysis

Total RNA was extracted using TRIzol reagent (Invitrogen, Carlsbad, CA, USA). The concentration of the solution was measured with a NanoDrop 1000 spectrophotometer (NanoDrop Technologies, Wilmington, DE, USA). The reverse transcription was then performed using the PrimeScript RT Master Mix (TaKaRa, Dalian, China) following the manufacturer's instructions. Each cDNA was denatured at 95°C for 5 min and amplified for 40 cycles: 15 s at 98°C, 30 s at 58°C, and 30 s at 72°C, using a LightCycler 96 (Roche, Basel, Switzerland). The mRNA expression level of each target gene was normalized to the respective *GAPDH* and analyzed. The qRT-PCR primer sequences are listed in Table 1. For each gene, independent experiments were carried out in triplicate.

### 2.8. Sodium Dodecyl Sulfate-Polyacrylamide Gel Electrophoresis (SDS-PAGE) and Western Blot Analysis

The cells were washed with ice-cold Dulbecco's phosphate-buffered saline (DPBS; HyClone, Logan, Utah, USA) three times followed by cellular lysis using lysis buffer (10 mM Tris-HCL, 1 mM EDTA, 1% sodium dodecyl sulfate, 1% Nonidet P-40, 1 : 100 proteinase inhibitor cocktail, 50 mM *β*-glycerophosphate, and 50 mM sodium fluoride) for 30 min. Protein quantification was performed by analyzing the absorbance at the 595 nm wavelength using a NanoDrop 1000 spectrophotometer (NanoDrop Technologies, Wilmington, DE, USA) with the Pierce® BCA Protein Assay Kit (Thermo Fisher Scientific, Germany). After boiling the samples (30 *μ*g protein/lane) for 10 min, the lysates were separated by 10%, 12%, or 15% SDS-PAGE and transferred to polyvinylidene fluoride (PVDF) membranes (Millipore, Temecula, CA, USA). The membranes were blocked for two hours using 5% (*w*/*v*) milk powder in PBST (PBS with 0.1% Tween) and incubated with the following primary antibodies overnight at 4°C: rabbit anti-human *α*7 nAChR (dilution 1 : 500; Abcam, Cambridge, UK), rabbit anti-human RUNX2 (dilution 1 : 2000; Abcam, Cambridge, UK), mouse anti-human RANKL (dilution 1 : 1000; Abcam, Cambridge, UK), mouse anti-human OPG (dilution 1 : 2000; Abcam, Cambridge, UK), rabbit anti-human ALP (dilution 1 : 2000; Abcam, Cambridge, UK), mouse anti-human GAPDH (dilution 1 : 3000; Boster Bio, Wuhan, Hubei, China), rabbit anti-human *β*-catenin (dilution 1 : 2000; Cell Signaling, Danvers, MA, USA) and rabbit anti-human GSK-3b (1 : 2000; Cell Signaling, Danvers, MA, USA), mouse anti-human active-*β*-catenin (1 : 1000; Millipore, Temecula, CA, USA), and mouse anti-human p-GSK-3b (1 : 2000; Santa Cruz, Dallas, Texas, USA).

Following three washes in TBST (Tris-buffered saline with 0.1% Tween, Proteintech Group, Wuhan, China), the membranes were incubated for two hours at room temperature with a horseradish peroxidase-conjugated anti-mouse or anti-rabbit IgG secondary antibody (Boster Bio, Wuhan, China). Immunodetection was performed on the PVDF membrane with an Odyssey infrared imaging system (LI-COR Biosciences, Lincoln, NE, USA).

### 2.9. Statistical Analysis

All experiments were performed in triplicate and data were handled using GraphPad Prism 6 (GraphPad Software, La Jolla, CA, USA). Data were expressed as the mean ± standard deviation (SD). Statistical analysis was performed using one-way analysis of variance (ANOVA) combined with the Tukey posttest. Statistical significance was determined for *P* < 0.05.

## 3. Results

### 3.1. Isolation, Culturing, and Identification of PDLSCs

The periodontally derived cells isolated from teeth of the UN, R, and P groups exhibited a typical spindle- and fibroblast-like appearance (Figure 1(a)). The cells were arranged in a radial-like distribution. As a result of osteogenic/adipogenic induction for 21 days and 28 days, respectively, the periodontally derived cells from each group formed mineralized nodules (alizarin red staining, Figure 1(b); marked by the black arrows) and lipid droplets (Oil red O staining, Figure 1(b)), indicating the multipotency of the isolated cells. The detection of surface molecule expression revealed that the periodontally derived cells were positive for mesenchymal markers, such as STRO-1, CD73, CD90, CD105, and CD146, while negative for the hematopoietic marker CD14, B cell marker CD19, endothelial marker CD34, pan-leukocyte marker CD45, and HLA-DR (Figure 1(c)) which characterized them as PDLSCs [21, 30, 31]; the overall expression patterns were similar among all groups, indicating PDLSCs remained a stem cell-like population.

### 3.2. Effects of Mechanical Pressure on Osteogenic and Osteoclastic Differentiation in PDLSCs

**Figure 2:**
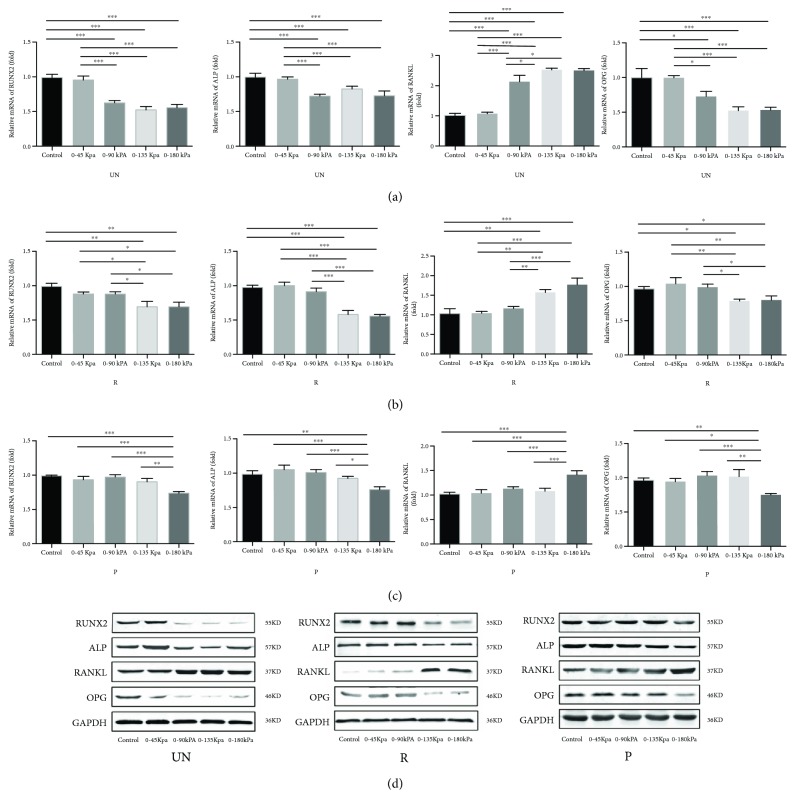
Effects of mechanical pressure on the osteogenic and the osteoclastic differentiation in PDLSCs. (a–c) qRT-PCR was performed to assess *RUNX2*, *ALP*, *RANKL*, and *OPG* expression levels in different groups. Values are reported as mean ± SD (^∗^*P* ≤ 0.05, ^∗∗^*P* ≤ 0.01, and ^∗∗∗^*P* ≤ 0.001; one-way ANOVA). (d) The expression levels of RUNX2, ALP, RANKL, and OPG in PDLSCs from each group were examined via Western blot assays. The cyclic hydraulic pressure was performed with a fixed frequency of 0.1 Hz at different ranges for 2 hours/day for three consecutive days.

The qRT-PCR assay indicated a significant reduction in the expression of *RUNX2*, *ALP*, and *OPG* in PDLSCs from the UN group (variable pressures at 0-90 kPa, 0-135 kPa, and 0-180 kPa), while the expression of *RANKL* was significantly enhanced under the same conditions, resulting in a significant increase in the *RANKL/OPG* ratio (*P* < 0.05, Figure 2(a)), indicating that certain hydrodynamic pressures could upregulate the *RANKL/OPG* ratio in PDLSCs. There were no significant differences among the study groups under pressures of 0-90 kPa, 0-135 kPa, or 0-180 kPa. Accordingly, similar trends of gene expression were detected in the R group (variable pressures at 0-135 kPa and 0-180 kPa, Figure 2(b)) and in the P group (at a pressure of 0-180 kPa only; Figure 2(c)), indicating that PDLSCs from different groups react differently to hydrodynamic pressure. Compared with the R group and the P group, the RANKL/OPG ratio was upregulated under lower pressure in the UN group. Moreover, the protein results of our Western blot assay were in accordance with the qRT-PCR assay (Figure 2(d)).

### 3.3. Effects of Mechanical Pressure on the Expression Levels of *α*7 nAChR in PDLSCs

**Figure 3:**
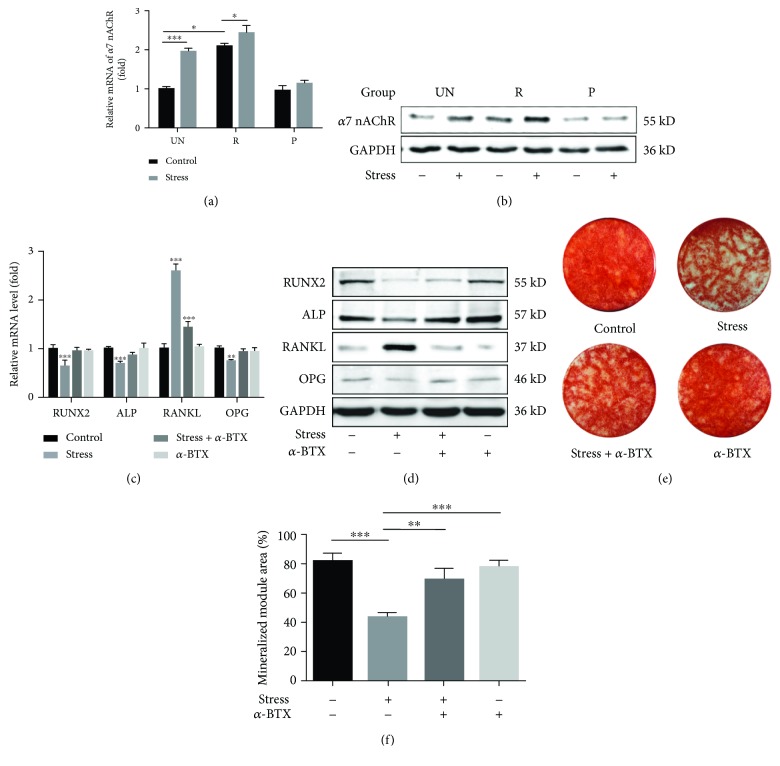
Mechanical pressure modulates the osteogenic and the osteoclastic differentiation through *α*7 nAChR in PDLSCs. (a) Relative mRNA expression of *α*7 nAChR in PDLSCs from different groups. (b) Western blot assays demonstrated the expression levels of *α*7 nAChR in PDLSCs from each group. (c) Relative mRNA expressions of *RUNX2*, *ALP*, *RANKL*, and *OPG* from UN group PDLSCs. (d) Western blot assay results showed the expression levels of RUNX2, ALP, RANKL, and OPG in PDLSCs from UN group PDLSCs. (e) Alizarin red staining of PDLSCs from each group after culture in osteogenic medium for 21 days under stereomicroscope. (f) Quantitative analysis for the mineralized nodule of (e). The cyclic hydraulic pressure was performed with a fixed frequency of 0.1 Hz at different ranges for 2 hours/day for three consecutive days (a–d) and 21 consecutive days while performing the osteogenic differentiation assays (e, f). Values have been expressed as mean ± SD. (^∗^*P* ≤ 0.05, ^∗∗^*P* ≤ 0.01, and ^∗∗∗^*P* ≤ 0.001; one-way ANOVA).

The expressions of *α*7 nAChR varied significantly in PDLSCs isolated from the three groups. As compared to the UN and P groups, the R group expressed a significantly greater level of *α*7 nAChR (*P* < 0.05; Figure 3(a)). Under hydrodynamic pressure of 0-135 kPa, the *α*7 nAChR expression was markedly upregulated in the UN group and slightly increased in the R group (*P* < 0.05). No statistically significant increase in the *α*7 nAChR expression was noted for the P group (Figures 3(a) and 3(b)). The expressions of RUNX2, ALP, and OPG were significantly reduced (*P* < 0.05) in PDLSCs from the UN group (pressure of 0-135 kPa), while the expression of RANKL was significantly enhanced, increasing the RANKL/OPG ratio. This tendency was in accordance with the *α*7 nAChR expression in the UN group but not in the R group. Specifically, treatment with an *α*7 nAChR inhibitor, *α*-BTX, reversed those results (Figures 3(c) and 3(d)). Alizarin red staining confirmed that PDLSCs formed fewer mineralized nodules following mechanical stress loading (Figures 3(e) and 3(f)). Furthermore, the *α*-BTX treatment completely reversed this effect (Figures 3(e) and 3(f)). These results indicated that mechanical stress regulated the expressions of RANKL, RUNX2, ALP, and OPG via *α*7 nAChR.

### 3.4. Wnt/*β*-Catenin Signaling Pathway Analysis of Genes in Response to Mechanical Stress

**Figure 4:**
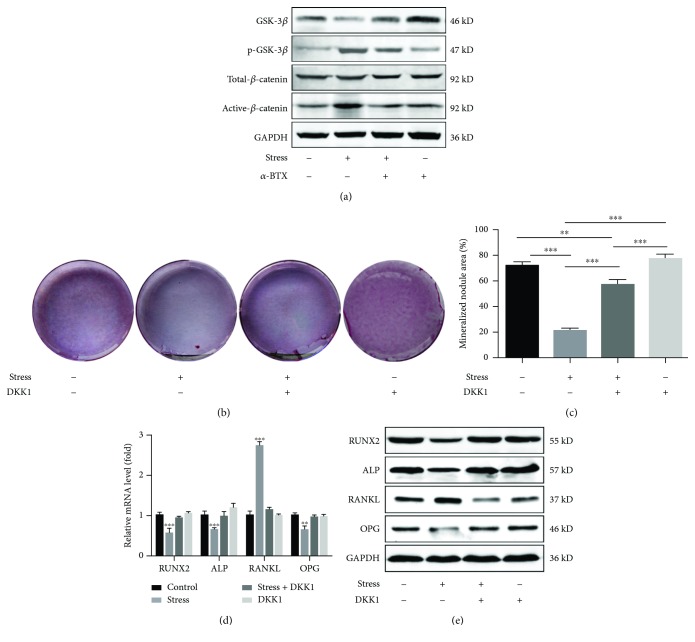
The Wnt/*β*-catenin signaling pathway analysis of genes in response to mechanical stress. (a) Western blot assays showing the expression levels of GSK-3*β*, p-GSK-3*β*, total-*β*-catenin, and active-*β*-catenin in the UN group PDLSCs. (b) Alizarin red staining of PDLSCs from all groups after culturing in the osteogenic medium for 21 days and the entire plate views of each group. (c) Quantitative analysis for mineralized nodule formation via alizarin red staining of PDLSCs from all groups. (d) Relative mRNA expressions of RUNX2, ALP, RANKL, and OPG from UN group PDLSCs. (e) The Western blot assay results show the expression levels of RUNX2, ALP, RANKL, and OPG in PDLSCs from UN group PDLSCs. The cyclic hydraulic pressure was performed with a fixed frequency of 0.1 Hz at different ranges for 2 hours/day for three consecutive days (a, d, and e) and 21 consecutive days (b, c). Values have been expressed as mean ± SD. (^∗^*P* ≤ 0.05, ^∗∗^*P* ≤ 0.01, and ^∗∗∗^*P* ≤ 0.001; one-way ANOVA).

Following the exposure of UN group cells to cyclic hydraulic pressure (0-135 kPa), Western blot results established a significant reduction in the expression of GSK-3*β*, while exhibiting a significant increase in the expression of p-GSK-3*β* and active-*β*-catenin (Figure 4(a)). There were no differences in the expression of total-*β*-catenin. The use of an *α*7 nAChR inhibitor, *α*-BTX, reversed the results (Figure 4(a)). These findings indicated that the altered expression of components of the Wnt signaling pathway was regulated by *α*7 nAChR under hydrodynamic pressure. Alizarin red staining showed that the PDLSCs formed fewer mineralized nodules following mechanical stress loading. The Wnt pathway inhibitor, DKK1, incompletely reversed this effect (Figures 4(b) and 4(c)). After treatment with DKK1, the qRT-PCR assay indicated that the expressions of *RUNX2*, *ALP*, and *OPG* were significantly increased (*P* < 0.05), while that of *RANKL* was significantly reduced (Figure 4(d)). The blocked Wnt/*β*-catenin pathways recovered the upregulation of osteoclastic differentiation and the RANKL/OPG ratio in PDLSCs following the mechanical stress loading. Moreover, the protein results of the Western blot assay were in accordance with the qRT-PCR data (Figure 4(e)). It can be suggested that the mechanical stress applied on the deciduous tooth PDLSCs can induce osteoclastic effects through upregulation of *α*7 nAChR and activation of the canonical Wnt pathway.

## 4. Discussion

The present study attempted to investigate the mechanism of PDLSC modulation on root resorption of human deciduous teeth under mechanical stress. For this purpose, PDLSCs were derived from deciduous and permanent teeth at different stages of root resorption. In order to simulate the physiologic effect of chewing forces, the PDLSCs were loaded with variable degrees of dynamic cyclic hydraulic pressure. Although a few studies used static pressure to mimic mechanical stress, the dynamic cyclic hydraulic pressure can better simulate the physiological chewing force. We prepared the cell pressure loading apparatus in our laboratory [26–28, 32] to simulate the complex stress loading conditions in the oral cavity. Hydraulic pressure can be maintained or gradually loaded using our apparatus. The pressure medium is isolated from air that prevents air dissolution in the culture medium and corresponding effects on the medium's pH and cultured cells. The range of mechanical stresses chosen in this study was based on finite element analysis by Poiate et al. [33] and our preliminary research. Although there are a number of advantages of the pressure loading apparatus, it could not fully simulate the real conditions. For instance, the bite force is pulse-like but not gradually fluctuating when chewing. Still, there is need to develop a new apparatus that can fully simulate the real chewing conditions.

The current study established that the expression of RANKL increased and the expressions of RUNX2, ALP and OPG decreased in each group of PDLSCs under certain mechanical stresses. As a result, the RANKL/OPG ratio was significantly increased. These findings suggested that varying degrees of mechanical stress at different root resorption extent of deciduous teeth are involved in the upregulation of the osteoclast differentiation by PDLSCs, which is consistent with the previous study conducted by Liu et al. who also reported that the force could regulate osteoclast differentiation by affecting the RANKL/OPG system in PDLSCs [34]. Zhang et al. reported that the RANKL/OPG ratio was reduced at 1 h and increased after 12 h in PDLSCs isolated from permanent teeth, while under static pressure [35]. Enough evidence exists suggesting that PDLSCs from permanent teeth presented osteogenesis activity under small static pressure or intense vibrations but osteoclastogenesis required a larger static pressure [16, 35, 36]. On the contrary, the current study did not show the osteogenesis of PDLSCs either from permanent teeth or deciduous teeth. The discrepancy among these studies is likely due to the different means of mechanical loading and the diversity of research cells.

Our results confirmed that PDLSCs in the UN group under variable pressures (0-90 kPa, 0-135 kPa, and 0-180 kPa) demonstrated a significant reduction in the expressions of RUNX2, ALP, and OPG. Interestingly, the expression of RANKL was significantly enhanced under similar conditions, thereby increasing the RANKL/OPG ratio. Similar changes were observed in the R group PDLSCs while being exposed to pressures at 0-135 kPa and 0-180 kPa and the P group PDLSCs at 0-180 kPa. These findings indicated that PDLSCs from the deciduous teeth at varying absorption periods react differently to mechanical stress. PDLSCs from unresorption teeth were seen as more sensitive to the applied force, while PDLSCs from permanent teeth expressed little to no sensitivity. Cordeiro et al. reported that the PDL from deciduous teeth had fewer epithelial cell rests of Malassez clusters and, correspondingly, significantly decreased expression of OPG compared to that seen with permanent teeth. Therefore, the deciduous teeth have lower protection against root resorption as compared to permanent teeth [37]. As a result of gradual increase in the chewing force coinciding with children's age, we may speculate that at some stage, the chewing forces lead to the increase of the RANKL/OPG ratio in PDLSCs. Finally, the osteoclastic process begins the physiologic root resorption of deciduous teeth.

Our investigation reported that the expression of *α*7 nAChR in PDLSCs from root resorption deciduous teeth was significantly higher compared to those from the nonresorption deciduous teeth and permanent teeth. These findings are consistent with a previous study [24]. The *α*7 nAChR is an important receptor for nicotine, expressed in neurons and periodontal tissues [38, 39]. Mandl et al.'s study demonstrated that nicotinic acetylcholine receptors may modulate osteoclastogenesis [19]. In addition, nicotine and *α*-BTX have been proven to regulate the expression of inflammatory cytokines, IL-1beta secretion, and RANKL expression [20]. As the expression of *α*7 nAChR was significantly higher during the active stage of root resorption, it can be speculated that the *α*7 nAChR plays an important role in physical root resorption. We found that the expression of the *α*7 nAChR was markedly upregulated in the UN group PDLSCs and only slightly increased in the R group PDLSCs under 0-135 kPa cyclic pressure for 3 days. It is suggestive that certain mechanical stresses are associated with the upregulation of the expressions of *α*7 nAChR in PDLSCs of deciduous teeth. Additionally, it was found that the osteoclastic effects influenced by cyclic pressure can be reversed using *α*-BTX. Hence, the mechanical stress may regulate the RANKL/OPG ratio and induce an osteoclastic effect in deciduous teeth through *α*7 nAChR. However, the *α*7 nAChR is a neuronal receptor and that is not a stress or pressure receptor [40]. Further research is required to explore the mechanism behind how mechanical stress can regulate the expression of *α*7 nAChR. Previously, it was reported that the mechanical stress can stimulate the expression of *α*7 nAChR in deciduous dental pulp stem cells [24]. As periodontal ligaments and dental pulp stem cells originate from different tissues, their association is still unclear.

In this study, we found that the expressions of active-*β*-catenin and p-Gsk-3*β* were upregulated under cyclic pressure. The changes were reversed while using the inhibitor *α*-BTX. An *in vivo* study revealed that the expression of active-*β*-catenin gradually increased within 3 days initially and decreased in response to the orthodontic force in PDL, during orthodontic tooth movement in rats [35]. Although we did not observe any decrease in the expression of active-*β*-catenin, it can be speculated that the canonical Wnt pathway can be activated by the regulation of the *α*7 nAChR under cyclic pressure. These findings are consistent with those of Zhou et al., where the *α*7 nAChR may negatively regulate the osteogenesis through the Wnt pathway in PDLSCs isolated from permanent teeth [21]. The Wnt pathway may play various roles under different conditions [41, 42]. Regard et al. uncovered evidence that the Wnt signaling pathway worked in bone development, remodeling, mechanotransduction, and fracture healing [41]. Furthermore, Zhang et al. [43] reported that the active Wnt pathway may lead to osteogenesis. Contrary results have been reported by Qin et al. [44] who reported that an activated Wnt pathway could lead to osteoclastogenesis. Although the activation of the Wnt/*β*-catenin pathway mostly correlated with osteogenesis of MSCs, there is no conclusion yet. Studies showed that activation of the canonical Wnt pathway inhibited the osteogenic differentiation of human PDLSCs in inflammatory microenvironments [45, 46]. Our previous study indicated that the occurrence and development of the physiological root resorption of deciduous teeth may be tightly associated with the inflammatory microenvironment [24]. These findings could explain our results in some ways. But further studies still need to prove the internal connection between the inflammatory microenvironment and root resorption of deciduous teeth.

We further investigated the inhibitor DKK1 and its ability to reverse the upregulation of RUNX2, ALP, and OPG and the negative regulation of the expression of RANKL under cyclic pressure. These findings support our hypothesis that cyclic pressure regulates the RANKL/OPG ratio and osteogenic differentiation through *α*7 nAChR regulation and the Wnt/*β*-catenin pathway in PDLSCs. Although the current study proved the effects of the Wnt/*β*-catenin pathway, the alizarin red staining results indicated that DKK1 could only partially reverse the osteoclastogenesis effects. These findings advocated the possible involvement of other cytokines or signal pathways regulating the osteoblastic/osteogenic activity during the root resorption process.

Ultimately, we found that mechanical stress may play a major role during the beginning of the physiological root resorption of deciduous teeth. The *α*7 nAChR participates in the root resorption process by regulating the Wnt/*β*-catenin pathway in PDLSCs. However, the hypothesis must be further validated in an ideal animal model, and the specific mechanism underlying how mechanical pressure promotes *α*7 nAChR expression must be explored.

## 5. Conclusions

The current study concluded that a certain cyclic pressure applied on deciduous tooth PDLSCs can induce osteoclastic effects through the upregulation of *α*7 nAChR and activation of the canonical Wnt pathway. This cascade leads to an increased expression of RANKL and decreased expressions of RUNX2, ALP, and OPG. It can be suggested that the chewing forces play a major role at the beginning of physiological root resorption of deciduous teeth.

## Figures and Tables

**Table 1:** The qRT-PCR primer sequences.

Target genes	Primer sequences	Accession number
*CHRNA7-F*	5′-GAG CCC TAC CCC GAT GTC A-3′	NM_000746
*CHRNA7-R*	5′-ATC TCA GCC ACG AGC A-3′	
*RUNX2-F*	5′-CAC TGG CGC TGC AAC AAG A-3′	NM_001024630
*RUNX2-R*	5′-CAT TCC GGA GCT CAG AAT AA-3′	
*ALP-F*	5′-CCT TGT AGC CAG GCC CAT TG-3′	NM_000478
*ALP-R*	5′-GGA CCA TTC CCA CGT CTT CAC-3′	
*RANKL-F*	5′-TGA TGT GCT GTG ATC CAA CGA-3′	NM_003701
*RANKL-R*	5′-AAG ATG GCA CTC ACT GCA TTT ATA G-3′	
*OPG-F*	5′-CAA AGT AAA CGC AGA GAG TGT AGA-3′	NM_002546
*OPG-R*	5′-GAA GGT GAG GTT AGC ATG TCC-3′	
*GAPDH-F*	5′-TCA TGG GTG TGA ACC ATG AGA A-3′	NM_002046
*GAPDH-R*	5′-GGC ATG GAC TGT GGT CAT GAG-3′	

Primer-BLAST (https://www.ncbi.nlm.nih.gov/tools/primer-blast/) was used for the primer design.

## Data Availability

The data used to support the findings of this study are available from the corresponding author upon request.
